# Public health implications of heavy metals in foods and drinking water in Ethiopia (2016 to 2020): systematic review

**DOI:** 10.1186/s12889-021-12189-3

**Published:** 2021-11-17

**Authors:** Dechasa Adare Mengistu

**Affiliations:** Department of Environmental Health, College of Health and Medical Science, Haramaya University, Harar, Ethiopia

**Keywords:** Public health, Heavy metals, Food safety, Drinking water, Health implication, Ethiopia

## Abstract

**Background:**

Besides their benefits, heavy metals are toxic, persistent, and hazardous to human health, even at their lower concentrations. Consumption of unsafe concentrations of food contaminated with heavy metals may lead to the disruption of numerous biological and biochemical processes in the human body. In developing country including Ethiopia, where untreated or partially treated wastewater is used for agricultural purposes, the problems related to the consumption foods contaminated with heavy metals may poses highest risk to human health. Therefore, this review was aimed to determine the public health implications of heavy metals in foods and drinking water in Ethiopia.

**Methods:**

The articles published from 2016 to 2020 were identified through systematic searches of electronic databases that include MEDLINE/PubMed, EMBASE, CINAH, Google Scholar, WHO, and FAO Libraries. The data was extracted using a predetermined data extraction form using Microsoft Excel, 2016. The methodological quality of the included studies was assessed using mixed methods appraisal tool (MMAT) version 2018 and Joanna Briggs Institute Critical Appraisal tools to determine the relevance of the studies. Finally, the results were evaluated based on the FAO/WHO guidelines for foods and drinking water.

**Results:**

A total of 1019 articles published from 2016 to 2020 were searched from various electronic databases and by manual searching on Google. Following the initial screening, 317 articles were retrieved for evaluation and 49 articles were assessed for eligibility, of which 21 studies were included in the systematic review. The mean concentration of Cr, Cd, Pb, As, Hg, Zn, Cu, Ni, Co, Fe and Mn in fruits and vegetables ranged from 2.068–4.29, 0.86–1.37, 1.90–4.70, 1.01–3.56, 3.43–4.23, 19.18–98.15, 4.39–9.42, 1.037–5.27, 0.19–1.0, 199.5–370.4, 0.26–869 mg/kg, respectively. The mean concentration Cr, Cd, Pb, As, Zn, and Fe in meat and milk ranged from 1.032–2.72, 0.233–0.72, 1.32–3.15, 0.79–2.96, 78.37–467.7, and 505.61–3549.9 mg/kg, respectively. The mean concentration of Cr, Cd, Pb, Zn, and Cu in drinking water ranged 0.0089–0.054, 0.02–0.0237, 0.005–0.369, 0.625–2.137, and 0.176–1.176 ml/L, respectively. The mean concentration of Cr, Cd, Pb, Zn, Cu, Ni, Co, Fe, and Mn in other edible cereals ranged from 0.973–2.165, 0.424–0.55, 0.65–1.70, 70.51–81.58, 14.123–15.98, 1.89–13.8, 1.06–1.59, 67.866–110.3, and 13.686–15.4 mg/kg, respectively.

**Conclusion:**

This systematic review identified heavy metals in foods and drinking water and determined their public health implications. The results of this finding imply that the majority of the studies reported high concentrations of toxic heavy metals in foods and drinking water that are hazardous to human health. Therefore, effective food safety and risk-based food quality assessment are essential to protect the public health.

**Supplementary Information:**

The online version contains supplementary material available at 10.1186/s12889-021-12189-3.

## Introduction

Heavy metals are metallic chemicals with a relatively high density that are toxic, persistent and hazardous to human health at low concentrations [1]. These include mercury (Hg), lead (Pb), copper (Cu), cadmium (Cd), arsenic (As), chromium (Cr), thallium (TI), manganese (Mn), zinc (Zn), and nickel (Ni) [2]. Some of these metals (Fe, Mn, Cu and Zn) are essential for metabolism in their lower concentrations [3]. As, Cd, Cr, Co, Pb, Ni, and Zn are the most common heavy metals potentially hazardous to human health [4]. However, cadmium and lead have more significant side effects on human health since they are easily accessible through the food chain [5, 6].

Consumption of unsafe concentrations of heavy metals in food may lead to the disruption of biological and biochemical processes in the human body [7]. These disorders are characterized by gastrointestinal disorders, stomatitis, tremors, diarrhea, hemoglobinuria, paralysis, vomiting, convulsions, and depression [8]. Similarly, heavy metals have the ability to disrupt metabolic activity and genetic makeup, or to affect embryonic or fetal development [9].

Currently, waste water is used for agricultural purposes in many countries of the world. At least 20 million hectares of land are irrigated with untreated or partially treated wastewater that poses the highest risk to the environment and human health [10, 11]. However, the problem is more severe in developing countries where reusing waste water for agricultural purposes is increasing from time to time [12]. This leads to the uptake and accumulation of various metals in foods and potential risks to human health [13, 14].

Therefore, food safety is an important public health issue, and is necessary to maintain food quality and to ensure that human beings are safe from food related health hazards [15, 16]. In many urban areas of Ethiopia, a large volume of untreated waste water is released into water bodies that are used for irrigation or agricultural purposes and has significant negative impacts on human health and the environment [17]. Besides these problems, there is no adequate country-wide knowledge base of the public health implications of heavy metals in foods and drinking water in Ethiopia.

Thus, this review aimed to determine the public health implications of heavy metals in foods and drinking water in Ethiopia that can be crucial to understand its implications, to take the appropriate measures by the concerned organizations, and to protect the public health.

### Eligibility criteria

The articles meeting the following criteria were included in the systematic review: -

- **Study area:** Research articles conducted in Ethiopia
- **Study design:** Cross-sectional studies
- **Language**: Articles published in English language
- **Population**: Articles conducted on any type of food and drinking water.
- **Publication issue**: Full text articles published in peer-reviewed journals from 2016 to 2020.
- **Outcome**: Articles reported the quantitative outcome of any heavy metals.

### Sources of information and search strategy

The original articles published from 2016 to 2020 were identified through the systematic searches of various electronic databases that include MEDLINE/PubMed, EMBASE, CINAH, Google Scholar, WHO, and FAO Libraries. Initially, the author (DA. Mengistu) conducted the search on the MEDLINE, followed by searching for the articles across the included electronic databases using the identified keywords, Medical Subject Headings (MeSH terms) and index terms. The following is a search term the author (DA. Mengistu) used in the initial search from PubMed**:** (((“Public health”[MeSH Terms] OR (“public”[All Fields] AND “health”[All Fields]) OR “public health”[All Fields]) AND (“implication”[MeSH Terms] OR “implication”[All Fields] OR “implications”[All Fields])) OR ((“public health”[MeSH Terms] OR (“public”[All Fields] AND “health”[All Fields]) OR “public health”[All Fields]) AND (“risk”[MeSH Terms] OR “risk”[All Fields] OR “risks”[All Fields])) OR ((“public health”[MeSH Terms] OR (“public”[All Fields] AND “health”[All Fields]) OR “public health”[All Fields]) AND hazards [All Fields])) OR ((“public health”[MeSH Terms] OR (“public”[All Fields] AND “health”[All Fields]) OR (“public health”[All Fields]) AND problems [All Fields])) AND ((“heavy metal”[MeSH Terms] OR (“heavy”[All Fields] AND “metal”[All Fields]) OR “heavy metal “[All Fields])) AND ((“food”[MeSH Terms] OR “food”[All Fields] OR (“drinking water”[MeSH Terms] OR (“drinking”[All Fields] AND “water”[All Fields]) OR “ drinking water”[All Fields])) AND (“Ethiopia”[All Fields]))).

The search strategy from **Embase** was as follows: (1) ‘public health’ OR ‘public health’/exp. OR ‘health’ OR ‘health’/exp. OR ‘implication’ OR ‘implication’/exp. OR ‘risk’ OR ‘risk’/exp. OR ‘heavy metal’ OR ‘heavy metal’/exp. OR ‘trace metal “food’ OR ‘food’/exp. OR ‘water supply’ OR ‘water supply’/exp. OR ‘drinking water’ OR ‘drinking water/exp’ OR ‘meat’ OR ‘meat’/exp. OR ‘fish’ OR ‘fish’/exp. OR ‘cereals’ OR ‘cereals’/exp.

Furthermore, manual searching of the articles was done to get additional studies relevant to this study.

### Study selection

All articles searched from various electronic databases were exported to ENDNOTE software version X5 (Thomson Reuters, USA). Duplicated articles were removed using the EndNote. Articles were screened by the author based on their titles and abstracts. Then, full-text articles were assessed against the inclusion criteria to determine their relevance to the study.

### Data extraction and quality assessment

The data were extracted from the included articles using a predetermined data extraction form, using Microsoft Excel 2016. The data regarding the author, year of publication, study area, study design, sample size, and outcome were extracted from the included articles. The extracted data was presented in the form of a table and text along with the main findings (concentration of heavy metals in foods and drinking water), types of foods, study location, and publication year.

The methodological quality of the included articles was evaluated using the mixed methods appraisal tool (MMAT) version 2018 [18] and Joanna Briggs Institute (JBI) Critical Appraisal tools [19]. These appraisal tools have the following nine evaluation criteria/ parameters; (1) appropriate sampling frame; (2) proper sampling technique; (3) adequate sample size; (4) study subject and setting description; (5) sufficient data analysis; (6) use of valid methods for the identified conditions; (7) valid measurement for all participants; (8) using appropriate statistical analysis, and (9) adequate response rate. The mean score was taken across all included studies and graded as high (80% and above score), moderate (60–80% score), and low (< 60% score) quality. Each included article was subjected to an evaluation (appraisal), at least three times at different time periods to check the accuracy of the work and to reduce errors. Finally, the articles meeting the inclusion criteria were included in the study.

### Outcome measures

The outcome of this systematic review aimed to determine the public health implications of heavy metals in foods and drinking water. The overall mean concentration of each heavy metal was calculated across the included articles. Finally, the concentration of each heavy metal was evaluated against Food and Agricultural Organization (FAO) and World Health Organization (WHO) guidelines developed for foods and drinking water.

## Results

### Study selection

**Fig. 1:**
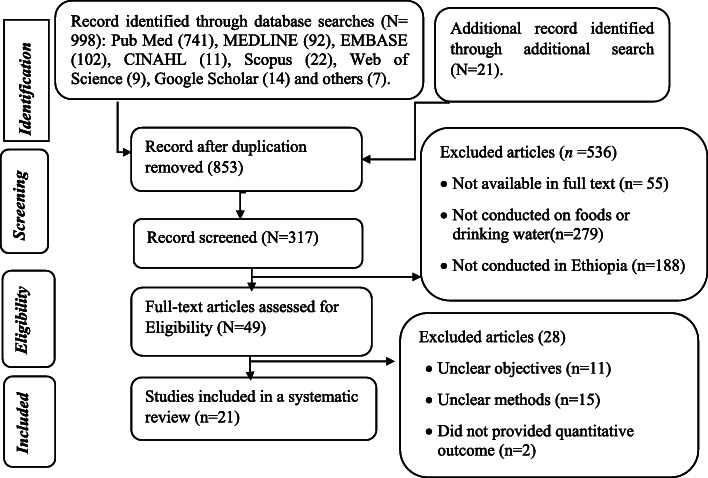
PRISMA flow diagram indicating the selection process of included articles for a systematic review, 2020

One thousand and nineteen published articles, abstracts, editorial papers, and reports were identified from various electronic databases. Nine hundred and ninety-eight articles were searched from electronic databases while 21 were searched manually from the Google. Among these articles, 741 were searched from PubMed, 92 from MEDLINE, 102 from EMBASE, 11 from CINAHL, 22 from Scopus, nine from the Web of Science, 14 from Google Scholar, seven articles from other electronic databases and 21 articles through manual searching. Following the initial screening, 317 articles were retrieved for evaluation and 49 articles were assessed for eligibility, of which 21 studies were included in the systematic review (Fig. 1).

### Characteristics of the included studies

A total of 21 articles conducted in Ethiopia and aimed to determine the concentration of heavy metals in any type of food and drinking water, and meet the inclusion criteria were included in the study.

Of 21 articles included in this systematic review, 6(28.57%) studies conducted in Oromia region [20–25], 5(23.8%) in Amhara region [25–29], 3 (14.29%) in Tigray [30–32], 2(9.5) %) in Southern Nations, Nationalities, and Peoples’ (SNNP) [33, 34], 1(4.76%) in Addis Ababa [35], 1(4.76%) in Harari [36], and 3 (14.29%) studies conducted in unspecific area (one in and around Addis Ababa [37]; one in Oromia and Addis Ababa [38] and one unspecified; conducted in Ethiopia) [39].

On the other hand, 5 (23.8%) studies conducted on vegetables alone [20, 21, 23, 36, 37], while one study included both fruit and vegetables [22]. Three (14.29%) and 3 (14.29%) studies were conducted to determine the concentration of heavy metals in drinking water [31, 32, 34] and fruit [26, 33, 39], respectively.

Furthermore, 2(9.5%) articles [25] were conducted to determine the concentration of heavy metals in fish tissue. Seven (33.3%) articles (one article on each of edible mushrooms [24]; milk [40]; honey [38]; barley [27]; popcorn and cornflakes [35]; sesame seeds [30]; and raw and roasted seeds and bread [28]) were conducted to determine the concentration of heavy metals in various raw and processed foods. Furthermore, among the included articles [20–40], 7 (33.3%) and 5 (23.8%) were published in 2020 [21, 26, 27, 29, 31, 33, 36] and in 2019 [20, 23, 25, 30, 32] respectively. (See Table 1 for details). All studies were cross-sectional studies. Almost all common toxic or hazardous heavy metals were included in the study. Similarly, the articles graded as high quality (having an 80% or above score) were included in this study.

**Table 1:** Overall characteristics of included articles, 2020

*Authors*	*Year*	*Region*	*Samples*	*Heavy metal concentration*	*Risk of bias*	*Reference*
Bahiru et al	2019	Oromia	Vegetable	The concentration of Cr, Cd and Pb in vegetable ranged from 2.90–3.77, 2.20–3.68 and 4.60–5.50 mg/kg, respectively.	Low	[20]
Gebeyehu and Bayissa	2020	Oromia	Vegetable	The concentration of As, Pb, Cd, Cr and Hg in vegetable ranged from 1.93–5.73, 3.63–7.56, 0.56–1.56, 1.49–4.63 and 3.43–4.23 mg/kg, respectively.	Low	[21]
Gezahegn et al	2017	In and around Addis Ababa	Leaf vegetables	The concentration of Zn, Cu, Ni, Co, Fe, Mn, Cr, As and Pb in leaf vegetables ranged from 10.9–219.3, 1.5–11.6, 0.7–11.6, 0.3–0.47, 40.8–193.6, 0.26–868.5, 1.7–8.8, 0.09–1.40 and 0.5–12.3 mg/kg, respectively.	Low	[37]
Marga	2016	Oromia	Fruit and vegetables	The concentration of Pb, Cd, Cu, Zn, Co and Ni in fruit and vegetables ranged from 0.14–0.31, ND-0.07, 1.29–6.44, 1.02–17.68, 0.08–1.54 and 0.39–2.20 mg/kg, respectively.	Low	[22]
Bahiru and Teju	2019	Oromia	Vegetable	The concentration of Fe, Zn and Cu ranged from 358.17–547.17, 45.63–62.46, and 10.20–15.07 mg/kg, respectively.	Low	[23]
Alamnie et al	2020	Harari	Green Leafy Vegetable	The mean concentration of Pb, Cd and Cr in vegetable were 0.17, 0.62 and 1.78 mg/kg, respectively.	Low	[36]
Haile et al	2018	Ethiopia (unspecified)	Wild edible plants (fruit)	The concentrations of Cu, Pb, Cd, Ni, and Cr in wild edible plants (fruit) were 4.57, 2.37, 0.93, 2.02, and 2.47 mg/kg, respectively.	Low	[39]
Babuskin et al	2020	SNNP	Fruit	The concentration of Zn, Cd, Cu, Co, Pb, Cr, Ni and Mn in fruit ranged from 1.3–6.3, ND-0.001, 0.44–6.2, 0.02–0.31, ND-0.008, ND-0.09, 0.12–8.8, 1.3–31 mg/kg, respectively.	Low	[33]
Adefa and Tefera	2020	Amhara	*Moringa oleifera*	The concentration of Cr, Cu and Zn in *moringa oleifera* was 6.675, 7.9 and 42.75 mg/kg respectively. Pb and Cd were below the detection limit.	Low	[26]
Shitahun and Tessema	2020	Amhara	Barley	The concentration of Zn, Fe, Ni, Mn, Cu, Cr, Co, Pb, and Cd in barley ranged from 33.7 to 76.7, 15.7 to 106, 3.78 to 27.6, 8.83 to 13.7, 5.83 to 10.5, 4.33 to 6.11, 3.38 to 5.83, 1.44 to 2.33, and 0.98 to 1.55 mg/kg, respectively.	Low	[27]
Abebe, et al	2017	Addis Ababa	Popcorn and cornflakes	The concentration of Cr, Mn, Fe, Co, Cu, Zn and Pb in the popcorn was 0.68, 6.17, 9.5, 1.41, 0.09, 88.3 and 0.94, respectively. The concentration of Cr, Mn, Fe, Co, Cu, Zn, and Pb in cornflakes was 0.30, 3.0, 5.5, 0.32, 0.30, 40.7 and 0.36 mg/kg, respectively. Cd and Ni were not ND.	Low	[35]
Gebrekidan & Desta	2019	Tigray	Sesame seeds	The concentration of Fe, Zn, Cu, Cd, and Pb in sesame seeds ranged from 35.5–43.1, 58.1–67.0, 15.3–21.3, 0.202–0.262, and 0.08–0.114 mg/kg, respectively.	Low	[30]
Abebe and Chandravanshi	2017	Amhara	Raw seeds and roasted seeds and bread	The concentration of Cr ranged from 0.17–1.58, 0.18–1.72 and 0.18–1.65; Mn from 1.04–3.98, 1.09–4.60 and 0.52–2.83; Fe from 18.0–115, 16.5–103, and 45.3–146; Co from 0.41–0.49, 0.50–0.76, and 0.34–0.75; Cu from 0.04–0.32, 0.04–2.72, and 0.05–3.12; Zn from 61.7–77.6, 59.2–83.0, and 108–116; Pb from 0.31–2.59, 0.82–3.11, 1.55–3.41 mg/kg for raw seed, roasted seed, and bread, respectively.	Low	[28]
Gebrelibanos et al	2016	Oromia	Edible Mushroom	The following concentration (mg/kg)of heavy metals was reported in edible mushroom (Pleurotus ostreatus); Cu (51.19), Fe (220.87), Zn (89.68) and Mn (47.55 mg/kg). The following concentration (mg/kg) of heavy metals was reported in edible mushroom (Pleurotus florida): Cu (53.56), Fe (243.92), Zn (95.26) and Mn (41.29 mg/kg). Cd and Pb were not detected.	Low	[24]
Kindie et al	2020	Amhara	Fish	The concentration (mg/kg) of As, Cd, Cr, Zn, and Fe in the muscle ranged from ND- 0.98, ND-0.19, 0.08–2.83, 6.53–627.08, and 14.23–164.77 mg/kg, respectively. Lead was not detected. The concentration (mg/kg) of As, Cd, Cr, Zn and Fe in the liver ranged from 1.58–4.94, 0.7–1.63, 2.65–6.12, 1.95–4.5, 227.38–769.67, and 997.0–6935.0 mg/kg, respectively.	Low	[29]
Gure et al	2019	Oromia	Fish	The concentration of Cr, Pd, Cd, Cu, and Co in fish tissue was 11.1, 7.57, 0.65, 7.7, and 4.1 mg/kg, respectively.	Low	[25]
Akele et al.	2017	Amhara	Milk	The concentration of Cr, Mn, Cu, Zn, Cd, and Pb in milk ranged from 0.468–0.828, 1.614–2.806, 0.840–1.532, 1.208–5.267, ND-0.330, and ND-0.186 mg/L, respectively.	Low	[40]
Haftu & Sathishkumar	2020	Tigray	Drinking Water	The concentration of Cd in drinking water ranged from 0.00125–0.011, Pb from 0.008–1.10, Cu from 0.515–3.515, Zn from 0.785–5.32, Cr from 0.015–0.15, Fe from 0.11–1.3, and Ni from 0.017–0.455 mg/L	Low	[31]
Desalegn et al	2018	SNNP	Drinking Water	The concentration of Cr in drinking water ranged from 0.004–0.006 mg/L. While Cr and Zn accounted 0.0036 and 0.599 mg/L, respectively. Cu, Pb, and Cd were not detected in drinking water.	Low	[34]
Ododo	2019	Tigray	Drinking Water	The concentration of Cd, Co, Cr, Cu, Fe, Mn, Ni, Pb, and Zn was 0.006, 0.015, 0.008, 0.013, 0.243, 0.228, 0.022, 0.007 and 0.492 mg/L, respectively.	Low	[32]
Yohannes et al	2018	Addis Ababa and Oromia	Honey	The concentration of Fe in honey samples ranged from 5.37–12.4, Ni from 0.80–4.46, Cr from 1.20–4.33, Zn from 1.92–4.22, Co from 0.60–1.17, Mn from 0.16–0.89, Cd from ND-0.69, and Cu from 0.09–0.47. Pb was not detected.	Low	[38]

*Keys*: *ND* Not Detected, *SNNP* Southern Nation Nationality and People

### Concentration of heavy metals in foods and drinking water

The mean concentration of Cr, Cd, Pb, As, Hg, Zn, Cu, Ni, Co, Fe and Mn in fruits and vegetables ranged from 2.068–4.29, 0.86–1.37, 1.90–4.70, 1.01–3.56, 3.43–4.23, 19.18–98.15, 4.39–9.42, 1.037–5.27, 0.19–1.0, 199.5–370.4, 0.26–869 mg/kg, respectively. The mean concentration Cr, Cd, Pb, As, Zn, and Fe in meat and milk ranged from 1.032–2.72, 0.233–0.72, 1.32–3.15, 0.79–2.96, 78.37–467.7, and 505.61–3549.9 mg/kg, respectively. The mean concentration of Cr, Cd, Pb, Zn, and Cu in drinking water ranged 0.0089–0.054, 0.02–0.0237, 0.005–0.369, 0.625–2.137, and 0.176–1.176 mg/L, respectively. The mean concentration of Cr, Cd, Pb, Zn, Cu, Ni, Co, Fe, and Mn in other types of foods ranged from 0.973–2.165, 0.424–0.55, 0.65–1.70, 70.51–81.58, 14.123–15.98, 1.89–13.8, 1.06–1.59, 67.866–110.3, and 13.686–15.4 mg/kg, respectively. Similarly, the study found the mean concentration of Cr, Cd, Pb, Zn, Cu, Ni, Co, Fe, and Mn ranged from 0.0012–0.0043, ND-0.0069, ND, 0.00192–0.00422, 0.00009–0.00047, 0.0008–0.00446, 0.0006–0.00117, 0.00537–0.0124, and 0.00016–0.00089 mg/kg, respectively (Table 1).

## Discussion

### Heavy metal concentration in foods and drinking water

Humans are exposed to heavy metals through various pathways [41]. Among these pathways, consumption of food contaminated with heavy metals is a major route for human exposure to heavy metals [42]. In the current study, the overall mean concentration of heavy metals was taken across the included studies, depending on the types of foods and drinking water. Besides their benefits, consumption of food contaminated with heavy metals may pose a risk to the health of humans. For example, chromium plays a major role in maintaining blood glucose levels within its recommended limits, but beyond that, it is toxic and hazardous to human health [43]. The current study found the mean concentration of Cr in fruits and vegetables, and drinking water ranged from 2.068–4.29 mg/kg, and 0.0089–0.054 mg/L, respectively that was higher than the maximum allowable limit of Cr in fruits and vegetables (2.3 mg/kg) [44], and drinking water (0.05 mg/L) [31, 45, 46]. This may be as the result of using an untreated or partially treated industrial waste water discharged to the environment which can contaminate the water supply.

Similarly, excessive lead exposure can cause adverse health effects such as hypertension, gastrointestinal effects, retarded growth, nervous system dysfunction, cognitive disability, hearing loss, and effects on reproduction [47]. However, the current study found the mean concentration of Pb in fruit and vegetables, edible cereals, fish and meat and drinking water ranged from 1.90–4.70 mg/kg, 0.65–1.70 mg/kg, 1.32–3.15 mg/kg, and 0.005–0.369 mg/L, respectively. These results were higher than the maximum allowable concentration of Pd in fruit and vegetables ranged from 0.05 to 0.3 mg/kg [44], 0.2 mg/kg in cereal grains [44], 0.3 mg/kg in fish [44], and 0.01 mg/L in natural water [48].

Cadmium accumulates in the human body, especially in the kidneys, and can damage the kidney [49]. The mean Cd concentration in drinking water ranged from 0.02–0.0237 mg/L, which was higher than the FAO/WHO, 2011 guidelines (0.003 mg/L) [44]. Furthermore, the mean concentration of Cd in fruit and vegetables ranged from 0.86–1.37 mg/kg, which was higher than the maximum permitted limit (0.05–0.1 mg/kg) [44].

Furthermore, the study found that the overall mean concentration of Cu in fruits and vegetables ranged from 4.39–9.42 mg/kg, which was lower than the maximum permissible level ranged from 4.5 mg/kg in fruit and [50] and 40 mg/kg in vegetables [44, 51]. Similarly, the concentration of Cu in drinking water ranged from 0.176–1.176 mg/L, which was lower than the maximum permissible limit (2.0 mg/kg) [44]. On the other hand, the study found the concentration of Cu in an edible mushroom ranged from 51.19 to 53.56 mg/kg, higher than the maximum permitted limit (0.05 to 5 mg/kg) [50, 52] and the mean concentration in fish accounted for 7.7 mg/kg, which was lower than the allowable limit (30.0 mg/kg) [53]. And also, the study found the mean concentration of Mn in fruit and vegetables ranged from 0.26–869 mg/kg that was lower than the guideline (500 mg/kg) [54].

An excess amount of iron in the body ‘s tissues adversely affect immune function, cell growth, and heart health [55, 56]. However, the current study found the mean concentration of Fe ranged from 199.5–370.4 mg/kg in fruits and vegetables that was lower than the allowable limit (425.5 mg/Kg) [54], and 505.61–3549.9 mg/kg in the meat and milk that was higher than the maximum allowable limit (100 mg/kg) [46, 57].

The maximum allowable concentration of Ni in fruit and vegetables ranged from 0.8 mg/kg for fruit [45] to 10 mg/kg for vegetables [44, 51]. Similarly, the current study found the mean concentration of Ni in fruit and vegetables ranged from 1.037–5.27 mg/kg that was within the standard limit. However, the mean concentration of Ni in drinking water was 0.019 to 0.24 mg/L that was higher than guidelines, 0.07 mg/L [31, 44].

Zinc is another metal which plays a vital role in the metabolic and physiological processes of many organisms and is important for growth and bone development. However, higher concentrations of Zn can cause poisoning in humans [31]. The current study found the overall mean concentration of Zn in fruit and vegetables ranged from 19.18–98.15 mg/kg that was less than the FAO/WHO guideline (99.4 mg/kg). The concentration of Zn in fish and meat was ranged from 78.37–467.7 mg/kg that was higher than the allowable limit standard (75 mg/kg), and Zn in edible mushrooms (89.68 to 95.26 mg/kg) higher than the FAO/WHO, 2004 guideline (0.3 to 1 mg/kg) and in drinking water ranged from 0.625–2.137 mg/L that was higher than the guidelines (0.2 mg/L).

The higher concentration of heavy metals in foods and drinking water in Ethiopia may be due to the rise in waste discharged from industrial activities such as paper mill, textile, and leather industries, and use of untreated or partially treated waste water for agricultural purposes.

In general, the concentration of most heavy metals in foods and drinking water was higher than the maximum allowable limit that would be a health risk to the consumer associated with the consumption of these foods and drinking contaminated water. Therefore, we recommend a strict regulatory control on the safety of foods and industrial waste to be discharged to the environment as well as to be used for agricultural purposes.

### Limitations

The review was based on previous studies that were conducted in different time periods. Therefore, the distribution may be incorrect. However, attempts were made to include all published articles on microbial quality and public health risk of ready-to-eat foods. Some important findings like conference proceedings and dissertations were not included in this study because of the type of search strategy adopted in this systematic review.

## Supplementary Information

**Additional file 1.**

## Data Availability

All data are included in the systematic review.

## Abbreviations

FAO: Food and Agricultural Organization
JBI: Joanna Briggs Institute
MeSH: Medical Subject Headings
MMAT: Mixed methods appraisal tool
PRISMA: Preferred Reporting Items for Systematic Reviews and Meta-Analysis
SNNP: Southern Nation Nationality and People
WHO: World Health Organization
